# 602 Delayed in Specialized Acute Burn Care in a Single Hospital in Puerto Rico

**DOI:** 10.1093/jbcr/iraf019.231

**Published:** 2025-04-01

**Authors:** Margarita Ramos, Luis De jesus III Vega, Jorge Pagan, Gabriel Medina, Jailenne Adorno De Gracia, Ana Rivera- Rivera

**Affiliations:** Wilma Vazquez Hospital; Univesity of Health Sciences Antigua; Fundacion A-Mar; Barry Univesity; Fundación A-Mar para Niños Quemados; Saint Luke’s Hospital

## Abstract

**Introduction:**

Puerto Rico currently faces a critical gap in burn care resources and specialized professional personnel. Despite a population of 3.2 million people, there is no centralized burn care facility on the island, and only four healthcare professionals are certified in Advanced Burn Life Support (ABLS). Moreover, there is a shortage of burn-fellowship trained surgeons. US national guidelines recommend one to two burn surgeons per 500,000 to 1 million people and at least one burn center per 1 million people to adequately address burn care needs, but Puerto Rico falls significantly short of these standards of care.

On top of this, there is little to no research documenting the burn incidence in Puerto Rico, though it is likely to be higher than in the mainland U.S. This lack of research and centralized resources directly impacts the timely care and recovery of burn patients, who often face delays in obtaining both acute treatment and necessary reconstructive surgery. Non profit organizations are left with the burden of addressing these gaps to improve the quality of burn care in Puerto Rico.

**Methods:**

This retrospective cohort study included 51 patients who visited a single hospital for burn care between February 2023 and September 2024. Statistical analysis was conducted using SPSS (v28). Demographic data, including age, gender, and hometown, were recorded, along with burn size (TBSA), time to initial healthcare visit, and time to evaluation by a burn surgeon. For patients who developed hypertrophic scars, the study recorded whether they received reconstructive treatment, and the type of treatment—split thickness skin graft (STSG), full thickness skin graft (FTSG), laser, or Kenalog injections.

**Results:**

The cohort consisted of patients with a mean age of 40-49 years, with 54.9% identified as female. Most patients were from the municipalities of San Juan, Vega Baja, Dorado, and Bayamon. The mean burn size was recorded at 3% of the total body surface area. On average, patients consulted a burn surgeon 6.3 days post-injury, with one visit other healthcare provider prior to burn surgeon specialist consultation. Among the 4 patients presenting with hypertrophic scars, 3 (75%) underwent burn reconstruction procedures: 3 received Kenalog injections, and 1 were not treated.

**Conclusions:**

There is a delay in acute burn care, with no correlation to burn size. There is also a delay to access burn surgeons of 6 days, demonstrating a need to improve timely access to specialized burn care. Most patients visited at least one health care facility prior to receiving definitive burn care. Limitations of this study include small sample size, referral bias, and retrospective nature of data.

**Applicability of Research to Practice:**

Delay in burn care is observed in Puerto Rico and there is a need to centralized burn care and increase the number of burn specialists in the island to improve access to timely burn care. Better triage of burn patients is needed as well as increased burn care education in Puerto Rico.

**Funding for the Study:**

N/A

